# Increased Amygdala Activations during the Emotional Experience of Death-Related Pictures in Complicated Grief: An fMRI Study

**DOI:** 10.3390/jcm9030851

**Published:** 2020-03-20

**Authors:** Manuel Fernández-Alcántara, Juan Verdejo-Román, Francisco Cruz-Quintana, Miguel Pérez-García, Andrés Catena-Martínez, María Inmaculada Fernández-Ávalos, María Nieves Pérez-Marfil

**Affiliations:** 1 Mind, Brain and Behavior Research Center (CIMCYC),University of Granada, 18071 Granada, Spain; mfernandeza@ua.es (M.F.-A.); fcruz@ugr.es (F.C.-Q.); mperezg@ugr.es (M.P.-G.); acatena@ugr.es (A.C.-M.); nperez@ugr.es (M.N.P.-M.); 2 Department of Health Psychology, University of Alicante, 03690 Alicante, Spain; inmaculada.fernandez@ua.es; 3 End-of-Life Research Network (EOL), 18071 Granada, Spain; 4 Laboratory of Cognitive and Computational Neuroscience (UCM-UPM), Centre for Biomedical Technology (CTB), 28223 Madrid, Spain

**Keywords:** grief, emotions, functional Magnetic Resonance Imaging, amygdala, reward, prefrontal cortex, putamen, death

## Abstract

Complicated grief (CG) is associated with alterations in various components of emotional processing. The main aim of this study was to identify brain activations in individuals diagnosed with CG while they were observing positive, negative, and death-related pictures. The participants included 19 individuals with CG and 19 healthy non-bereaved (NB) individuals. Functional magnetic resonance imaging (fMRI) scans were obtained during an emotional experience task. The perception of death-related pictures differed between the CG group and the NB group, with a greater activation in the former of the amygdala, putamen, hypothalamus, middle frontal gyrus, and anterior cingulate cortex. Amygdala and putamen activations were significantly correlated with Texas Revised Inventory of Grief scores in the CG group, suggesting that the higher level of grief in this group was associated with a greater activation in both brain areas while watching death-related pictures. A significant interaction between image type and group was observed in the amygdala, midbrain, periaqueductal gray, cerebellum, and hippocampus, largely driven by the greater activation of these areas in the CG group when watching death-related pictures and the lower activation when watching positive-valence pictures. In this study, individuals with CG showed significantly distinct brain activations in response to different emotional images.

## 1. Introduction

The death of a loved one triggers emotional reactions that are generally natural and adaptive. However, these manifestations are excessively intense and prolonged in around 10% of cases, leading to persistent or complicated grief (CG) [1,2]. CG is characterized by: Intense feelings of yearning for the deceased; great difficulty in accepting the loss; emotional symptoms such as anger, guilt, sadness, or feelings of emptiness; emotional numbness; and rumination [3,4,5].

CG has been associated with a lesser expressiveness of emotions [6], difficulties in the flexible regulation of emotions [7,8], a subjective emotional experience pattern of more unpleasant feelings [9], and a distinct processing of stimuli with a strong emotional component or directly associated with the loss of a loved one. In this regard, studies on approach-avoidance responses to reminders of the loss found that individuals with CG respond more slowly when faced with death-related words [10,11] and take longer to avoid images associated with death or loss [12]. In addition, greater attentional avoidance of emotional stimuli related to the deceased was observed in individuals with high levels of rumination [13,14], who were also found to respond more slowly to negative or positive emotional stimuli not directly related to their loss [15,16]. In this line, Freed et al. [17] studied avoidance and intrusiveness in an emotional Stroop task using death-related words versus neutral words. They found that activation of the dorsal part of the amygdala and the middle frontal gyrus was correlated with avoidance and that the ventral part of amygdala and the cingulate gyrus were correlated with intrusiveness [17].

Various fMRI studies have reported altered emotional processing after the death of a loved one. Gündel et al. [18] exposed grieving individuals to photographs and words related to the deceased and found brain activation in areas involved in emotional regulation and autobiographical memory. The same experimental paradigm was used to compare between individuals with CG versus normal grief, finding greater activation in the nucleus accumbens (NA) of those with CG [19]. The NA is involved in reward, salience, motivation, decision-making, and inhibitory control [20], and it is considered central to the development and maintenance of different types of addiction. In a later study, Arizmendi et al. used an emotional counting Stroop task to evaluate emotional regulation in an elderly population [21]. They reported a significantly greater activation in the rostral portion of the anterior cingulate cortex, with an activation peak in the right orbitofrontal cortex, in participants with normal grief in comparison to those with CG or not bereaved, who did not show this activation pattern. However, they found activation in the dorsal portion of the anterior cingulate cortex in the CG group during the last phase of the experimental task.

Limitations of fMRI studies of grieving individuals to date include the absence of a CG diagnosis in some investigations (e.g., [18,22,23]). Furthermore, the response to affective autobiographical stimuli related to the deceased person is frequently compared with the response to a neutral condition alone (e.g., [18,19,23]). Although exposure to this type of material appears to increase emotional reactivity [24], behavioral studies have also observed emotional alterations in response to other types of pleasant or unpleasant stimuli in individuals with CG [6,9,16]. It is therefore of interest to identify neural correlates of emotional processing by individuals with CG in response to other stimuli besides those related to their loss, using emotional pictures with different valence and arousal ratings [25,26,27]. To the best of our knowledge, this is one the first studies to evaluate whether death-related pictures activate similar brain areas to those activated in response to other types of negative valence pictures in a population with complicated grief.

With this background, the main objective of this study was to compare brain activation in response to pictures belonging to three categories (pleasant/positive valence, unpleasant/negative valence, and death-related) between a CG group and a non-bereaved (NB) group. A secondary objective was to explore the interaction between the brain activation evoked by each type of picture and group membership and its association with measures of grief intensity. There were two study hypotheses: (1) In comparison to the NB group, the CG group would show greater activation in the amygdala, in frontal areas, and in areas associated with the reward circuit when viewing death-related pictures, based on studies by Arizmendi et al. [21], Freed et al. [17] and O’Connor et al. [19]; and (2) the CG group would show less activation in areas associated with the processing of pleasant stimuli (limbic and subcortical areas) when viewing positive pictures, with a significant interaction of group with this type of image and death-related pictures, based on studies by Diminich & Bonanno [6] and Fernández-Alcántara et al. [9].

## 2. Material and Methods

### 2.1. Participants

Participants were recruited from the Psychology Clinic of the School of Psychology (Granada, Spain), the Palliative Care Unit of the San Cecilio University Hospital (Granada, Spain), grief associations (Alma y Vida in Jaén, Spain), and from responders to advertisements in different media. Inclusion criteria for the CG group were: (a) Aged over 18 years; (b) loss of a loved one in the previous 18 months; (c) diagnosis with CG based on an ad-hoc clinical interview and score > 25 in the Inventory of Complicated Grief (ICG); and (d) absence of other psychopathologies according to the SCL-90-R or Global Assessment of Post-Traumatic Stress Disorder (initials in Spanish, EGEP) questionnaire. Inclusion criteria for the NB group were: (a) Aged over 18 years; (b) no loss of a loved one in the previous three years; (c) no CG symptoms according to clinical interview or ICG score; and (d) values below the 25th percentile of SCL-90-R or EGEP scores for anxiety, depression, and post-traumatic stress (compared with the general population sample). Exclusion criteria for both groups were: (a) Aged over 65 years; (b) habitual or frequent consumption of illicit substances; (c) the presence of neurodegenerative disease, (d) failure to meet the requirements for MRI in the safety questionnaire, and (e) the presence of gross anatomical abnormalities observed in the brain images by an expert radiologist.

Out of an initial CG sample of 56 grieving participants, 37 were excluded for habitual substance use (*n* = 2), medical problems (*n* = 4), or absence of CG/high psychopathological scale score (*n* = 31). The final study sample therefore comprised 19 participants in the CG group and 19 participants matched for sociodemographic characteristics who were included in the NB group (see Table 1). All participants were right-handed except for one member of the CG group, who was ambidextrous.

### 2.2. Procedure

In the first evaluation session, written informed consent was first obtained from the participants, followed by an interview and self-report measures. Participants meeting criteria for one of the two study groups then underwent a second 90-minute session that included an MRI study and evaluation of the affective stimuli. At the end of the MRI session, each participant was given time to talk and receive emotional support. These sessions were conducted at the Center for Research, Mind, Brain, and Behavior (Centro de Investigación, Mente, Cerebro y Comportamiento, CIMCYC) by one of two researchers (MFA and MIFA) under identical conditions. The study was approved by the Committee of Ethics in Human Research of the University of Granada and is part of a research project financed by the Campus of International Excellence (CEI Biotic; Reference: CEI2014-MPBS34).

### 2.3. Instruments

#### 2.3.1. ICG (Inventory of Complicated Grief)

This instrument was developed by Prigerson et al. [28] and adapted into Spanish by Limonero-García et al. [29]. The 19-item ICG evaluates experiences of the major symptoms of CG over the previous 4 weeks: Longing for the deceased, rumination, emotional aspects, and hallucinations. The internal consistency of this scale has been high across studies, reaching values of Cronbach’s α = 0.94.

#### 2.3.2. TRIG (Texas Revised Inventory of Grief)

This inventory comprises 21 items divided into two subscales (Past Behavior and Present Feelings) and assesses the past and current intensity of emotional symptoms related to grief [30], with responses on a 5-point Likert scale (0–4). Lower scores indicate higher grief intensity. Cronbach’s α values of 0.75 and 0.86 have been obtained in Spanish populations.

#### 2.3.3. Symptoms Checklist (SCL-90-R)

This inventory of 90 symptoms created by Derogatis [31] comprises 90 items that evaluate a wide range of psychopathologies. The depression and anxiety subscales were used in the present study. The reliability indices (Cronbach’s α) of the different scales range from 0.81 to 0.90.

#### 2.3.4. Global Assessment of Post-Traumatic Stress Disorder (EGEP in Spanish)

This instrument was created by Crespo and Gómez [32] to evaluate different components of post-traumatic stress disorder and contains three sections that assess: Previous history of traumatic events; severity/intensity of symptoms; and daily functioning. The present study used only the second section, with 62 items that evaluate the dimensions of re-experimentation, avoidance, and hyperactivity associated with the experience of a traumatic event (in this case, the death of a loved one). The internal consistency (Cronbach’s α) of the different subscales range from 0.73 to 0.86 [32].

#### 2.3.5. Emotional Experience Task in Functional MRI (fMRI)

This task includes pictures with different affective contents (pleasant/positive valence, unpleasant/negative valence, and death-related). Pictures from the International Affective Picture System (IAPS) were used for the negative and positive valence conditions, [33,34], excluding any pictures related to death or fatal accidents in the negative valence condition. Pictures from the death-scheme database were selected for the death-related condition [26], representing cemeteries, body parts (associated with fatal accidents), terminal illnesses, and accidents, among others. A control condition was also included, in which a black fixation cross was displayed in the center of the screen. A total of 15 pictures were used for each condition. Arousal values were adjusted a priori among the three conditions (F (2.42) = 2.04, *p* = 0.143) as were valence values between the death and negative valence conditions (t (28) = 1.32, *p* = 0.196) (Supplementary Material, S1). The task was programmed with E-Prime software [35] using a block design with the same presentation order for all participants, displayed on a resonance-compatible screen through an inverted mirror system. Three pictures of each condition were used in each block, and each image was displayed for 5 seconds (15 seconds for each block). The fixation cross was displayed for 15 seconds (equivalent to one block). A random image-distribution method based on a Latin square was used to counter-balance conditions across the task. The task had a total duration of 10 minutes.

Imaging Data Acquisition and Preprocessing: MRI data were acquired on a 3 Tesla Magnetom Tim Trio scanner (Siemens Medical Solutions, Erlangen, Germany) at the Mind, Brain and Behavior Center (CIMCYC) of the University of Granada, using a 32-channel receive-only head coil. During the task, a T2*-weighted echo-planar imaging (EPI) sequence was acquired with the following parameters: repetition time (TR), 2000 ms; echo time (TE), 30 ms; flip angle, 70°; field of view (FOV), 240 mm; number of slices, 33; voxel dimension, 3 × 3 × 3 mm; gap, 1 mm; n° volumes, 300. Images were collected axially and parallel to the AC–PC plane. A sagittal three-dimensional T1-weighted image was also obtained in the same session to rule out gross anatomical abnormalities, using the following parameters: TR, 8 ms; TE, 4 ms; flip angle, 8°; FOV, 240 mm; number of slices, 160; voxel dimension, 1 × 1 × 1 mm.

Functional images were preprocessed with Statistical Parametric Mapping (SPM8) software (Wellcome Department of Cognitive Neurology, Institute of Neurology, Queen Square, London) run on Matlab R2009 (MathWorks, Natick, MA, USA). Preprocessing included: Re-slicing to the mean image of the time series, normalization to an EPI template in the Montreal Neurobiological Institute (MNI) space, and spatial smoothing by convolution with a 3D Gaussian kernel (full width at half maximum (FWHM) = 8 mm). Data were high-pass filtered to remove low-frequency noise (1/128 Hz) and corrected for temporal autocorrelation using an autoregressive AR model. Data from one participant of the CG group were excluded due to excessive movement (>2 mm) during the fMRI task.

#### 2.3.6. Picture Recognition Using the IAPS

After the fMRI protocol, participants completed the picture-recognition task in an adjacent laboratory, identifying the pictures that they had viewed during the previous resonance task. In addition, 90 pictures (30 each in the negative valence, positive valence, and death-related conditions) were randomly presented in a task designed using E-Prime software, including 45 from the fMRI task alongside 45 others with the same content, valence, and arousal values. After viewing each picture, the participant indicated whether it had appeared during the fMRI task. The total percentage of correct answers was recorded.

#### 2.3.7. Subjective Emotional Evaluation of Affective Pictures via the Self-Assessment Manikin (SAM)

After the recognition task, the subjective emotional experiences of participants were assessed in relation to the 45 pictures viewed during the fMRI task. Assessments used the SAM scale, a self-report pictographic measure to evaluate the three dimensions of an emotional response proposed by Lang [36]: valence, arousal, and dominance.

### 2.4. Data Analysis

#### 2.4.1. Behavioral Analyses

SPSS version 22 (Chicago, IL, USA) was used to analyze the behavioral data. Between-group differences in demographic and task-related variables were evaluated with chi-squared tests and t-tests for independent samples for categorical and continuous variables, respectively.

#### 2.4.2. Neuroimaging Analyses

Task regressors were convolved with the SPM8 canonical hemodynamic response function. Four task regressors (positive, negative, death-related images, and fixation cross) were modeled for the 15 seconds that the images of each block were screened. Three contrasts of interest were defined in the first-level analysis: (i) Death pictures > Fixation cross, (ii) Negative pictures > Fixation cross, and (iii) Positive pictures > Fixation cross. The resulting first-level contrast images were then used in second-level random-effect analyses to assess between-group differences. Two-sample t-tests were used to compare the groups in the three contrasts of interest. In addition, a 2 (Group) × 3 (Image type) full factorial analysis was performed to assess the interaction between the brain activation evoked by each type of picture and group. Given the significant differences in depression scores, these values were included as covariate in all neuroimaging analyses.

#### 2.4.3. Threshold Criteria

The imaging results were corrected for multiple comparisons using the Alphasim thresholding approach implemented in SPM REST toolbox [37]. This method is one of the most used in neuroimaging [38,39,40]. To determine whether a cluster of significant results is corrected for multiple comparisons, the Alphasim tool run Monte Carlo simulations to calculate the minimum cluster extent required based in a previously selected *p*-value. These simulations are computed using several inputs, (i.e, a brain mask, a previously selected individual voxel *p*-value, a cluster connection radius and the smoothness of data). Further details of the method can be found elsewhere [41].

For within-groups one-sample t-tests, Alphsim input parameters included a whole brain mask of 229781 voxels, an individual voxel threshold probability of *p* < 0.001, and a cluster connection radius of 5 mm, considering the smoothness of data after model estimation. A minimum cluster extent (KE) of 340, 325, and 343 voxels for contrasts 1, 2, and 3 respectively, were estimated corresponding to a corrected *p* value < 0.05. Between-group comparisons for each contrast were masked by the results of basic activation and deactivation maps derived from the corresponding one-sample t-tests. A minimum KE of 51, 41, and 33 were defined for contrast 1, 2, and 3 respectively. Interaction results were masked by the sum of the significant activation and deactivation maps of the three contrasts. A KE of 73 voxels (within a mask of 62873) was estimated.

## 3. Results

### 3.1. Behavioral Results

Participants recognized most of the pictures displayed during the emotional fMRI task (95.95% for the CG group and 98.63% for the NB group), with no significant between-group differences (*p* > 0.05). No between-group differences were found in valence, arousal, or dominance values (see Table S1 in Supplementary Material).

### 3.2. Neuroimaging Results

Within-group activations for each contrast are reported in Supplementary Material (see Tables S2, S3 and S4 in Supplementary Material).

#### 3.2.1. fMRI Main Task Effects

##### Death Pictures > Fixation Cross

**Figure 1:**
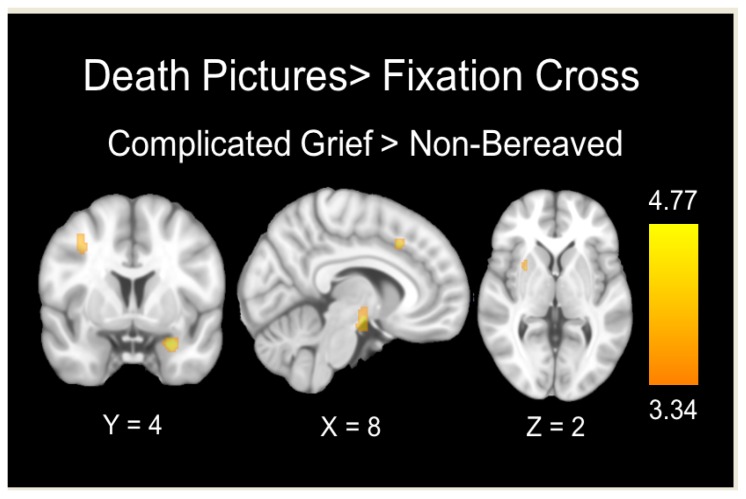
Brain regions showing significant between-group differences during the processing of death-related pictures. The color bar indicates t-values.

In the contrast of death-related pictures with the fixation cross, between-group comparisons revealed significantly increased activation in the CG versus NB group in the left amygdala, hypothalamus, right lateral middle frontal gyrus, right putamen, and dorsal anterior cingulate cortex extending to the supplementary motor area. Conversely, no region showed significantly increased activation in the NB versus CG group (Table 2 and Figure 1).

##### Correlation Analyses

Amygdala and putamen activations in the CG group significantly correlated with the TRIG-Present scores in the CG group (*r* = −0.570, *p* = 0.013; *r* = −0.490, *p* = 0.039, respectively) but not in the NB group (*r* = 0.169, *p* = 0.489; *r* = 0.181, *p* = 0.457, respectively); i.e., a higher level of present grief in the CG group was associated with a greater activation of both brain areas while watching death-related pictures. Furthermore, activation of the middle frontal gyrus was correlated with the TRIG- Past scores in the CG group (*r* = 0.522, *p* = 0.026) but not in the NB group (*r* = 0.014, *p* = 0.955).

##### Negative Valence Pictures > Fixation Cross

**Figure 2:**
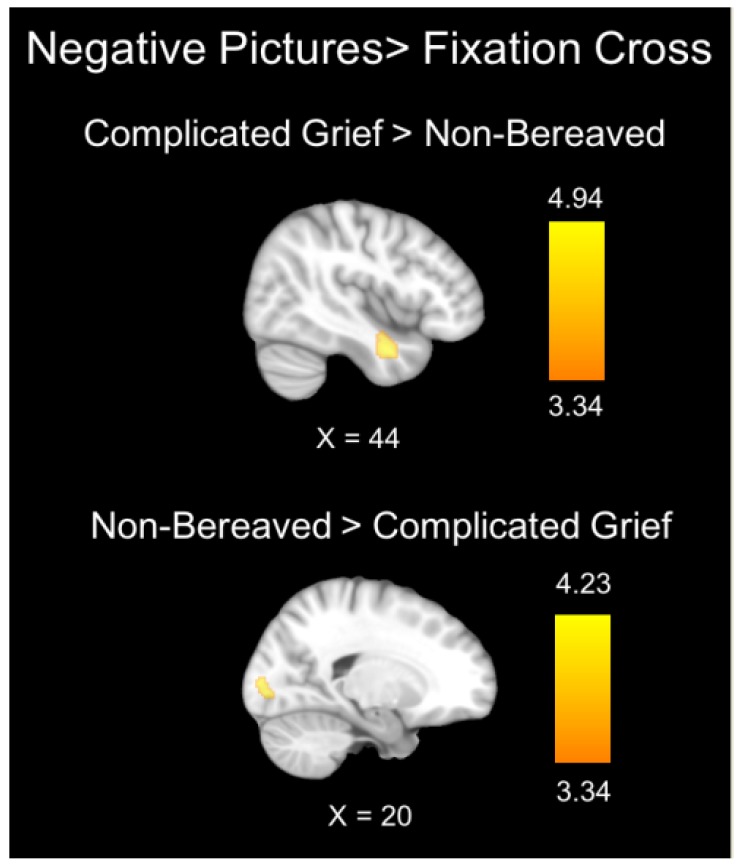
Brain regions showing significant between-group differences during the processing of negative valence pictures. The color bar indicates *t*-values.

In the contrast of negative valence pictures with the fixation cross, between-group comparisons showed significantly greater activation in the right inferior temporal cortex in the CG versus NB group and significantly greater activation in both the association and primary visual cortices in the NB versus CG group (Table 3 and Figure 2).

##### Positive Valence Pictures > Fxation Cross

No significant between-group differences were found at the selected threshold during the viewing of positive pictures in comparison to the fixation cross.

#### 3.2.2. Interaction Analysis

##### Death x Negative Valence

Analysis of the interaction between death-related and negative pictures by group did not reveal significant results at the selected threshold.

##### Death x Positive Valence

**Figure 3:**
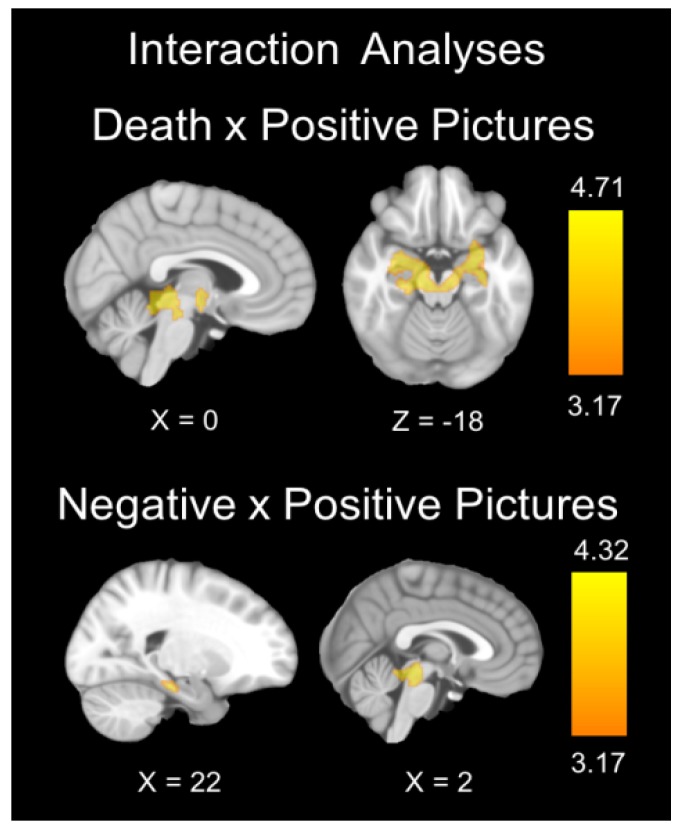
Brain regions showing significant group x image type interaction. The color bar indicates *t*-values.

**Figure 4:**
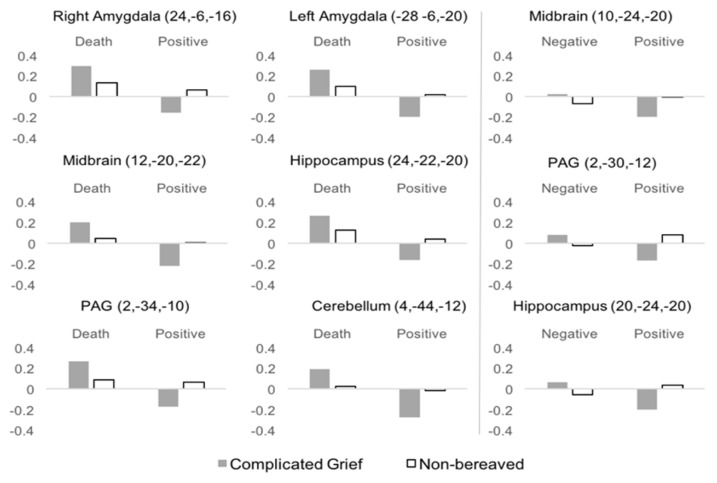
Mean activations for each group and image type in regions showing significant interaction. Left and Right panels represent significant results of the Death x Positive, and Negative x Positive interactions respectively.

Analysis of the interaction between death-related and positive pictures by group showed significant results in the amygdala, midbrain, periaqueductal gray matter, cerebellum, and right hippocampus. All of these interactions are mainly attributable to the greater activation and greater deactivation of these regions in the CG group during the presentation of death-related and positive pictures, respectively (Table 4, Figure 3 and Figure 4).

##### Positive Valence x Negative Valence

Analysis of the interaction between positive versus negative valence pictures by group showed significant results in the midbrain, periaqueductal gray matter, and right hippocampus, mainly due to the greater activation and greater deactivation of these regions in the CG group during the viewing of negative and positive pictures, respectively (Table 4, Figure 3 and Figure 4).

## 4. Discussion

The aim of this investigation was to identify brain activations associated with emotional processing in individuals diagnosed with CG when faced by three different categories of affective pictures: Positive valence, negative valence, and death-related. The results supported the first study hypothesis, indicating greater activation in the amygdala, frontal areas (right lateral middle frontal gyrus), and reward-circuit areas (right putamen) in the CG group while viewing death-related pictures. In addition, TRIG-Present subscales were positively correlated with both amygdala and putamen activations in the CG group, and the TRIG-Past subscale was positively correlated with middle frontal gyrus activation. The results also supported the second hypothesis, revealing a significant interaction between groups in the contrast of positive-valence pictures and death-related pictures. These interactions were driven by the greater activation of the amygdalae, midbrain, PAG, cerebellum, and right hippocampus in the CG group while viewing death-related pictures and the greater deactivation of these regions in the same group while viewing positive valence pictures.

In relation to the first hypothesis, previous studies have highlighted the role of the amygdala in processing death-related stimuli [17,42] and its interaction with prefrontal areas involved in emotional regulation processes [43,44]. A recent study found a greater connectivity between the amygdala and the ventrolateral prefrontal cortex in individuals with high versus low self-esteem, supporting this hypothesis [45]. With respect to the putamen, various studies have described its greater activation, along with that of the insula, during the processing of affective death-related words versus words with positive or negative valence [25,46]. The higher activation of these areas in the CG while viewing death-related pictures may result from the greater emotional regulation needed to cope with this type of stimulus.

The correlation of amygdala and middle frontal gyrus activation with TRIG scores may indicate a more intense grief experience, including emotional responses (sadness/yearning), difficulties in accepting the death, or thoughts related to the deceased [47], is associated with a greater activation of the amygdala while viewing death-related pictures. Freed et al. [17] also reported that the amygdala and middle frontal gyrus were associated with avoidance behaviors in grieving persons exposed to death-related stimuli. However, these associations changed when death-related pictures were replaced with photographs of the deceased, observing a significant correlation between NA activation and the degree of yearning for the loved one [19]. Interestingly, no association was found with the ICG, suggesting that these activations may not be related to the CG symptoms gathered by this instrument.

Regarding the activation of frontal areas, several investigations have reported their significantly greater activation in grieving individuals. Specifically, hyperactivation was found in the orbitofrontal areas of individuals who had experienced a breakup [48,49] and in the frontal gyrus of those suffering the death of a loved one [18] or the loss of pet [17]. Individuals with perinatal grief also showed hyperactivation of frontal areas while viewing pictures of healthy infants [50]. Recently, their activation was recorded during the processing of affective stimuli related to the deceased [21], and activation in the putamen, caudate nucleus, insula, and orbitofrontal cortex was observed when grieving persons were thinking about the deceased [23]. These areas are thought to be involved in the processing of economic gains and losses and in the management of rewards and reinforcers, among other functions [51]. The above findings support the proposition that individuals with CG show greater activation in the reward system during the emotional processing of death-related stimuli. In the present study, analysis of the interaction with death and negative pictures did not reveal significant results. One possible explanation is that the selected death pictures, which were matched for valence and arousal levels with the negative valence pictures, were considered along a continuum of unpleasant pictures within the participants’ emotional space [52]. In this case, death-related pictures were at the extreme of the emotional space and it was these that generated statistically significant differences, unlike the negative valence pictures. Conversely, a different pattern of differences was found during processing of negative valence images. The CG group showed increased activation of the inferior temporal cortex and decreased activation of primary and association visual cortices compared to the NB group. Weaker activation of visual areas in response to negative stimuli has been previously found in major depression disorder [53] and could be related to attentional bias to negative stimuli previously found in grieving populations [16]. Based in Gündel et al., the higher activation of the inferior temporal cortex suggests higher object-recognition areas in the context of grief [18].

The present results partially support the second hypothesis. Although no statistically significant group differences were found when comparing positive pictures with the fixation point, differences were found in the interactions with negative-valence and death-related pictures. Interactions were observed in reward circuit regions and in those associated with processing reinforcers (i.e., periaqueductal gray matter, midbrain, amygdala, and putamen). The CG group showed greater activation of these areas while processing death-related versus positive pictures, whereas the NB group show a similar brain pattern across conditions. This result might indicate a difference in the processing by individuals with CG between positive or pleasant emotional stimuli and death-related stimuli [6,9,16]. Although previous investigations have shown a greater activation of the reward system while viewing pictures of the deceased person [19], the present results suggest that this system is inversely altered while processing positive/pleasant affective stimuli. It is possible that individuals with CG may have a reward response to reminders of the loss [19] but not to other affective and positive stimuli. This may explain some of the clinical characteristics of CG, especially the difficulties in experiencing a positive connection with other people and in engaging with positive and pleasant emotions [10].

Taken together, the present results indicate that death-related pictures trigger higher activity in frontal and subcortical areas of individuals with CG. They also reveal a different processing of positive-valence pictures in comparison to the non-bereaved. Death-related pictures evoked cortical activations in both groups, but they were more intense among those with CG. More research is needed to clarify the role of pleasurable or positive stimuli in relation to grief; however, these results are consistent with previous findings on emotional decision-making with reinforcers [54,55] and on the difficulties associated with processing positive emotions [6,8,9].

With respect to the clinical implications of these findings, they appear to support bereavement interventions that emphasize acceptance of the reality of the loss (unpleasant aspects) and the envisioning of a future without the deceased [56]. According to the model developed by Maccallum and Bryant [10], CG symptoms persist because of attachment to the deceased and the inflexible use of emotional regulation strategies. Hence, the aim of interventions would be to change these inadequate beliefs and maladaptive emotional strategies, with greater emphasis on unlinking the merged identities that keep the individual ruminating on the deceased. The intervention model proposed by Boelen et al. [57] has the same objectives and considers that rumination on the circumstances of the death and behaving as if the deceased were alive are methods to avoid the reality of death.

Limitations of the present study include the small sample size of the groups, although the size of the CG group is among the largest in fMRI studies to date in comparison with previous studies [19,21], and a strict statistical correction was employed to guarantee the validity of the imaging results. In addition, it did not include individuals with normal grief. Moreover, researchers were not blinded regarding the diagnosis and group assignations. Finally, statistically significant differences were found between groups in the Past Behavior score of the TRIG, which may indicate that they initially differ in the baseline. Future studies should explore differences related to the intensity of grief among a CG group, NB group, and a “normal grief” group. A further limitation is that scores for depression were higher in the CG group than in the NB group, and CG is strongly associated with depression, anxiety, and/or PTSD. Nevertheless, depression levels were included as a covariate in all imaging analyses.

## 5. Conclusions

In conclusion, these results indicate that individuals with CG have a distinct emotional processing of different types of emotional stimuli. Death-related stimuli evoked an intense response in the CG group, with greater activation in the amygdala and middle frontal gyrus that positively correlated with their present experience of grief. Finally, the CG group also manifested a more intense response in the amygdala to death-related pictures compared with positive and pleasant pictures. Clinical interventions in cases of CG should address the role of complex unpleasant emotions and the experience of positive and pleasant states.

## Figures and Tables

**Table 1:** Sociodemographic data and emotional psychopathology levels.

Variables	CG (*n* = 19)	NB (*n* = 19)	t or χ^2^	*p*
	Mean (SE) or *n* (%)	Mean (SE) or *n* (%)		
**Age**	40.42 (2.74)	39.42 (2.85)	–0.25	0.802
**Sex**			0	1
Men	2 (10.5%)	2 (10.5%)		
Women	17 (89.5%)	17 (89.5%)		
**Marital status**			1.56	0.670
Single	8(42.1%)	8 (42.1%)		
Married	8 (42.1%)	10 (52.6%)		
Widow/widower	1 (5.3%)	0 (0%)		
Separated	2 (10.5%)	1 (5. 3%)		
**Education**			0	1
Basic	2 (10.5%)	2 (10.5%)		
Bachelor/FP	9 (47.4%)	9 (47.4%)		
University student	8 (42.1%)	8 (42.1%)		
**Time since loss (months)**	41.58 (5.51)			
**Drug consumption**			0.17	0.676
Yes	3 (15.8%)	4(21.1%)		
No	16 (84.2%)	15 (79%)		
**Relationship to deceased**				
Son	4 (21.1%)			
Spouse	1 (100%)			
Mother/father	12 (63.2%)			
Brother	1 (5.3%)			
Grandparent	1 (5.3%)			
Another relative	0 (0%)			
Friend	0 (0%)			
**Inventory of Complicated Grief (ICG)**	36.32 (1.67)	6.89 (1.58)	–12.76	< 0.001
**Texas Revised Inventory of Grief (TRIG)**				
Past Behavior	19.63 (1.29)	31.68 (1.65)	5.74	< 0.001
Present Feelings	24 (1.15)	53.26 (1.96)	12.86	< 0.001
**Depression (SCL-90-R)**	20.68 (2.13)	4.37 (0.95)	–6.99	< 0.001
**Anxiety (SCL-90-R)**	10.8 (1.30)	2.32 (0.59)	-5.94	< 0.001
**Post-traumatic stress (EGEP)**				
Symptoms of re-experimentation	3.68 (0.23)	0.32 (0.17)	–11.73	< 0.001
Intensity of re-experimentation symptoms	7.16 (0.75)	0.53 (0.31)	-8.20	< 0.001
Symptoms of avoidance—Affective bewilderment	2.95 (0.43)	0.05 (0.05)	–6.60	< 0.001
Intensity of avoidance symptoms—affective bewilderment	6.58 (1.23)	0.05 (0.05)	–5.31	< 0.001
Symptoms of hyper-activation	3.37 (0.31)	0.21 (0.14)	–9.30	< 0.001
Intensity of hyper-activation symptoms	6.63 (0.99)	0.37 (0.28)	–6.06	< 0.001

Note. CG = complicated grief; NB = non-bereaved control group; SE = Standard Error.

**Table 2:** Brain regions showing significant between-group differences during the processing of death-related pictures.

Brain Region	X	Y	Z	kE	t-Value
CG > NB					
Amygdala	−26	2	−22	55	4.77
Hypothalamus	−2	−8	−8	337	4.16
Lateral middle frontal gyrus	38	6	44	52	4.33
Putamen	28	8	2	64	3.45
Dorsal anterior cingulate	4	22	40	69	3.77

Note. CG = complicated grief group; NB = non-bereaved control group, x,y,z = peak MNI coordinates; kE= Cluster extent in voxels.

**Table 3:** Brain regions showing significant between-group differences during the processing of unpleasant/negative valence pictures.

Brain Region	X	Y	Z	kE	*t*-Value
CG > NB					
Inferior temporal cortex	44	0	26	107	4.94
NB > CG					
Visual association cortex	−40	−78	2	81	4.23
Primary visual cortex	18	−90	−2	127	4.06

Note. CG = complicated grief group; NB = non-bereaved control group x,y,z = peak MNI coordinates; kE = Cluster extent in voxels.

**Table 4:** Brain regions showing significant interaction between types of emotional picture by group.

Brain Region	X	Y	Z	kE	t Value
Death x positive interactions					
Amygdala	24	−6	−16	1 916 ^a^	3.92
Amygdala	−28	−6	−20	1 916 ^a^	4.35
Midbrain	12	−20	−22	1 916 ^a^	4.71
Hippocampus	24	−22	−20	1 916 ^a^	3.60
Periaqueductal gray	2	−34	−10	1 916 ^a^	4.21
Cerebellum	4	−44	−12	1 916 ^a^	4.54
Negative x positive interactions					
Midbrain	10	−24	−20	423 ^b^	4.17
Periaqueductal gray	2	−30	−12	423 ^b^	4.11
Hippocampus	20	−24	−20	423 ^b^	4.32

Note. CG = complicated grief group; NB = non-bereaved control group x,y,z = peak MNI coordinates; kE = Cluster extent in voxels; ^a,b^ = part of the same cluster.

## Supplementary Materials

The following are available online at https://www.mdpi.com/2077-0383/9/3/851/s1, Table S1: Means and standard deviations for 45 emotional pictures used in the fMRI task., Table S2: Brain regions showing significant activation or deactivation associated with the processing of death-related pictures., Table S3: Brain regions showing significant activation or deactivation associated with the processing of unpleasant/negative valence pictures. Table S4: Brain regions showing significant activation, deactivation, or between-group differences associated with the processing of pleasant/positive valence pictures.

## Data Sharing Statement

The datasets generated during and/or analyzed during the current study are available from the corresponding author on reasonable request.

## References

[1] Lundorff M., Holmgren H., Zachariae R., Farver-Vestergaard I., O’Connor M. (2017). Prevalence of prolonged grief disorder in adult bereavement: A systematic review and meta-analysis. J Affect. Disord..

[2] Prigerson H.G., Vanderwerker L.C., Maciejewski P.K., Stroebe M.S., Hansson R.O., Schut H., Stroebe W. (2008). Prolonged Grief Disorder: A Case for Inclusion in DSM-V. Handbook of Bereavement Research and Practice: Advances in Theory and Intervention.

[3] American Psychiatric Association (2014). Diagnostic and Statistical Manual of Mental Disorders (DSM-5®).

[4] Arizmendi B., O′Connor M.F. (2015). What is “normal” in grief?. Aust. Crit. Care.

[5] Bryant R.A. (2014). Prolonged grief: Where to after Diagnostic and Statistical Manual of Mental Disorders. Curr. Opin. Psychiatry.

[6] Diminich E.D., Bonanno G. (2014). Faces, Feelings, Words: Divergence across Channels of Emotional Responding in Complicated Grief. J. Abnorm. Psychol..

[7] Bonanno G.A., Papa A., Lalande K., Westphal M., Coifman K.A. (2004). The importance of being flexible: The ability to both enhance and suppress emotional expression predicts long-term adjustment. Psychol. Sci..

[8] Gupta S., Bonanno G. (2011). Complicated grief and deficits in emotional expressive flexibility. J. Abnorm. Psychol..

[9] Fernández-Alcántara M., Cruz-Quintana F., Pérez-Marfil M.N., Catena-Martínez A., Pérez-García M., Turnbull O.H. (2016). Assessment of emotional experience and emotional recognition in complicated grief. Front. Psychol..

[10] Maccallum F., Bryant R.A. (2013). A cognitive attachment model of prolonged grief: Integrating attachments, memory, and identity. Clin. Psychol. Rev..

[11] O′Connor M.F., Arizmendi B. (2014). Neuropsychological correlates of complicated grief in older spousally bereaved adults. J. Gerontol. B Psychol. Sci. Soc. Sci..

[12] Maccallum F., Sawday S., Rinck M., Bryant R.A. (2015). The push and pull of grief: Approach and avoidance in bereavement. J. Behav. Ther. Exp. Psychiatry.

[13] Eisma M.C., Shut H.A.W., Stroebe M.S., Van den Bout J., Stroebe W., Boelen P.A. (2014). Is Rumination after Bereavement Linked with Loss Avoidance? Evidence from Eye-Tracking. PLoS ONE.

[14] Eisma M.C., Rinck M., Stroebe M.S., Schut H.A.W., Boelen P.A., Stroebe W., Van den Bout J. (2015). Rumination and implicit avoidance following bereavement: An approach avoidance task investigation. J. Behav. Ther. Exp. Psychiatry.

[15] Delespaux E., Zech E. (2015). Why do bereaved partners experience interfering rumination? Evidence for deficits in cognitive inhibition. Death Stud..

[16] Delespaux E., Zech E. (2017). Deficits in cognitive inhibition and post-loss rumination: Evidence from a thought suppression task/Déficits de inhibición cognitiva y rumiación posterior a una pérdida: Evidencia a partir de una tarea de supresión de pensamientos. Estud. Psicol. Stud. Psychol..

[17] Freed P.J., Yanagihara T.K., Hirsch J., Mann J.J. (2009). Neural mechanisms of grief regulation. Biol. Psychiatry.

[18] Gündel H., O’Connor M.F., Littrell L., Fort C., Lane R.D. (2003). Functional neuroanatomy of grief: An FMRI study. Am. J. Psychiatry.

[19] O’Connor M.F., Wellisch D.K., Stanton A.L., Eisenberger N.I., Irwin M.R., Lieberman M.D. (2008). Craving love? Enduring grief activates brain’s reward center. NeuroImage.

[20] Volkow N.D., Wang G.J., Tomasi D., Baler R.D. (2013). Obesity and addiction: Neurobiological overlaps. Obes. Rev..

[21] Arizmendi B., Kaszniak A.W., O′Connor M.F. (2016). Disrupted prefrontal activity during emotion processing in complicated grief: An fMRI investigation. NeuroImage.

[22] O’Connor M.F., Gündel H., McRae K., D’Lane R. (2007). Baseline Vagal Tone Predicts BOLD Response during Elicitation of Grief. Neuropsychopharmacology.

[23] Schneck N., Haufe S., Tu T., Bonanno G.A., Ochsner K., Sajda P., Mann J.J. (2017). Tracking Deceased-Related Thinking with Neural Pattern Decoding of a Cortical-Basal Ganglia Circuit. Biol. Psychiatry Cogn. Neurosci. Neuroimaging.

[24] Salas C.E., Radovic D., Turnbull O.H. (2012). Inside-out: Comparing internally generated and externally generated basic emotions. Emotion.

[25] Han S., Qin J., Ma Y. (2010). Neurocognitive processes of linguistic cues related to death. Neuropsychologia.

[26] Martí-García C., García-Caro M.P., Cruz-Quintana F., Schmidt-RioValle J., Perez-Garcia M. (2016). Emotional Responses to Images of Death A New Category of Emotional Processing?. OMEGA J. Death Dying.

[27] Martí-García C., Fernández-Alcántara M., Schmidt-RioValle J., Cruz-Quintana F., García-Caro M.P., Perez-Garcia M. (2017). Specific emotional schema of death-related images vs unpleasant images/Esquema emocional específico de imágenes relacionadas con la muerte frente a imágenes desagradables. Stud. Psychol. Estud. Psicol..

[28] Prigerson H.G., Maciejewski P.K., Reynolds C.F., Bierhals A.J., Newsom J.T., Fasiczka A., Miller M. (1995). Inventory of Complicated Grief: A scale to measure maladaptive symptoms of loss. Psychiatry Res..

[29] Limonero-García J., Lacasta-Reverte M., García J., Maté-Méndez J., Prigerson H.G. (2009). Adaptación al castellano del inventario de duelo complicado. [Spanish adaptation of the inventory of complicated grief]. Med. Paliativa.

[30] García-García J., Landa-Petralanda V., Trigueros-Manzano M., Gaminde-Inda I. (2005). Inventario Texas Revisado de Duelo (ITRD): Adaptación al castellano, fiabilidad y validez [Texas Revised Inventory of Grief (TRIG): Adaptation to Spanish, reliability and validity]. Aten. Primaria.

[31] Derogatis L.R. (2002). SCL-90-R: Cuestionario de 90 Síntomas. [SCL-90-R: Symptom Checklist-90-R: Administration, Scoring, and Procedures Manual].

[32] Crespo M., Gómez M. (2012). La evaluación del estrés postraumático: Presentación de la escala de Evaluación Global de Estrés Postraumático (EGEP). [Posttraumatic Stress Assessment: Introducing the Global Assessment of Posttraumatic Stress Questionnaire]. Clínica Salud.

[33] Lang P.J., Bradley M.M., Cuthbert B.N. (1990). Emotion, attention, and the startle reflex. Psychol. Rev..

[34] Vila J., Sánchez M., Ramírez I., Fernández M.C., Cobos P., Rodríguez S., Moltó J. (2001). El sistema internacional de imágenes afectivas (IAPS): Adaptación española. Segunda parte. [The international affective picture system (IAPS): Spanish validation. Second part]. Rev. Psicol. Gen. Apl..

[35] Schneider W., Eschman A., Zuccolotto A. (2002). E-Prime: User’s Guide.

[36] Lang P.J. (1995). The emotion probe: Studies of motivation and attention. Am. Psychol..

[37] Song X.W., Dong Z.Y., Long X.Y., Li S.F., Zuo X.N., Zhu C.Z., He Y., Yan C.G., Zang Y.F. (2011). REST: A toolkit for resting-state functional magnetic resonance imaging data processing. PLoS ONE.

[38] Dennis E.L., Gotlib I.H., Thompson P.M., Thomason M.E. (2011). Anxiety modulates insula recruitment in resting-state functional magnetic resonance imaging in youth and adults. Brain Connect..

[39] Huang F.Y., Hsu A.L., Hsu L.M., Tsai J.S., Huang C.M., Chao Y.P., Hwang T.J., Wu C.W. (2019). Mindfulness improves emotion regulation and executive control on bereaved individuals: An fMRI study. Front. Hum. Neurosci..

[40] Bueso-Izquierdo N., Verdejo-Román J., Contreras-Rodríguez O., Carmona-Perera M., Pérez-García M., Hidalgo-Ruzzante N. (2016). Are batterers different from other criminals? An fMRI study. Soc. Cogn. Affect. Neurosci..

[41] Ward B.D. (2013). AFNI and NIFTI Server at NIMH in Bethesda, MD, USA. Simultaneous Inference for FMRI Data. http://afni.nimh.nih.gov/pub/dist/doc/manual/AlphaSim.pdf.

[42] Quirin M., Loktyushin A., Arndt J., Küstermann E., Lo Y.Y., Kuhl J., Eggert L. (2012). Existential neuroscience: A functional magnetic resonance imaging investigation of neural responses to reminders of one’s mortality. Soc. Cogn. Affect. Neurosci..

[43] Banks S.J., Eddy K.T., Angstadt M., Nathan P.J., Phan K.L. (2007). Amygdala–frontal connectivity during emotion regulation. Soc. Cogn. Affect. Neurosci..

[44] Kim M.J., Loucks R.A., Palmer A.L., Brown A.C., Solomon K.M., Marchante A.N., Whalen P.J. (2011). The structural and functional connectivity of the amygdala: From normal emotion to pathological anxiety. Behav. Brain. Res..

[45] Yanagisawa K., Abe N., Kashima E.S., Nomura M. (2016). Self-esteem modulates amygdala-ventrolateral prefrontal cortex connectivity in response to mortality threats. J. Exp. Psychol. Gen..

[46] Shi Z., Han S. (2012). Transient and sustained neural responses to death-related linguistic cues. Soc. Cogn. Affect. Neurosci..

[47] Futterman A., Holland J.M., Brown P.J., Thompson L.W., Gallagher-Thompson D. (2010). Factorial validity of the Texas Revised Inventory of Grief—Present scale among bereaved older adults. Psychol. Assess..

[48] Fisher H.E., Brown L.L., Aron A., Strong G., Mashek D. (2010). Reward, Addiction, and Emotion Regulation Systems Associated With Rejection in Love. J. Neurophysiol..

[49] Najib A., Lorberbaum J.P., Kose S., Bohning D.E., George M.S. (2004). Regional brain activity in women greiving a romantic relationship breakup. Am. J. Psychiatry.

[50] Kersting A., Ohrmann P., Pedersen A., Kroker K., Samberg D., Bauer J., Kugel H., Koelkebeck K., Steinhard J., Heindel W. (2009). Neural activation underlying acute grief in women after the loss of an unborn child. Am. J. Psychiatry.

[51] Rushworth M.F.S., Noonan M.P., Boorman E.D., Walton M.E., Behrens T.E. (2011). Frontal cortex and reward-guided learning and decision-making. Neuron.

[52] Bradley M.M., Lang P.J., Lane R.D., Nadel R. (2000). Measuring Emotion: Behavior, Feeling and Physiology. Cognitive Neuroscience of Emotion.

[53] Li J., Xu C., Cao X., Gao Q., Wang Y., Wang Y., Peng J., Zhang K. (2013). Abnormal activation of the occipital lobes during emotion picture processing in major depressive disorder patients. Neural Regen. Res..

[54] Fernández-Alcántara M., Pérez-García M., Pérez-Marfil M.N., Catena-Martínez A., Hueso-Montoro C., Cruz-Quintana F. (2016). Assessment of different components of executive function in grief. Psicothema.

[55] Maccallum F., Bonanno G.A. (2016). The economics of losing a loved one: Delayed reward discounting in prolonged Grief. Clin. Psychol. Sci..

[56] Maccallum F., Bryant R.A. (2011). Imagining the future in complicated grief. Depress. Anxiety.

[57] Boelen P.A., van den Bout J., van den Hout M.A. (2006). Negative cognitions and avoidance in emotional problems after bereavement: A prospective study. Behav. Res. Ther..

