# Supplementary material for: Increased Amygdala Activations during the Emotional Experience of Death-Related Pictures in Complicated Grief: An fMRI Study
Source: J Clin Med. 2020 Mar 20;9(3):851. doi: 10.3390/jcm9030851 (PMC7141501; doi:10.3390/jcm9030851)
Supplement: Supplementary file 1 [file jcm-09-00851-s001.pdf]

Supplementary Material

Table S1. Means and standard deviations for 45 emotional pictures used in the fMRI task.

Category	Picture code	Valence M (SD)	Arousal M (SD)	Dominance M (SD)
Death	Graveyard (x8); Cadaver (x2); Terminal disease (x3); Accident (x2)	2.27(0.37)	5.56 (0.60)	5.52 (0.44)
Negative Valence	2682; 2692; 2722; 2751; 2752; 3180; 6010; 6821; 9041; 9080; 9120; 9290; 9415; 9530; 9560;	2.47(0.48)	5.61 (0.91)	3.41 (0.82)
Positive Valence	1463; 2010; 2299; 2346; 2360; 4614; 4622; 5260; 7220; 7502; 8190; 8380; 8461; 8496; 8600	7.68(0.42)	5.04 (0.91)	5.78 (0.64)

Note: M = Mean, SD = Standard Deviation.

Neuroimaging within group results

Death pictures > fixation cross

In the contrast of death pictures with the fixation cross, both groups showed bilateral activation in the occipital cortex, intraparietal cortex, and fusiform gyri. The CG group also showed activation in the amygdala, hippocampus giri, thalamus, anterior insula, orbitofrontal cortices, middle frontal giri, supplementary motor area, anterior cingulate cortex, and right putamen. The NB group (but not CG group) showed significant deactivation of areas of the default mode network (i.e., medial prefrontal cortex and angular gyrus), the anterior cingulate cortex, and the superior temporal cortices extending to the posterior insula (see Table SM2).

Table S2. Brain regions showing significant activation or deactivation associated with the processing of death-related pictures.

Brain region	CG					NB				
	X	Y	Z	kE	t value	X	Y	Z	kE	t value
Death pictures > Fixation Cross										
Activations										
Occipital lobe	16	-88	-10	23 102[a]	7.91	22	-92	6	23 112[a]	11.64
Occipital lobe	-14	-90	-8	23 102[a]	7.53	-40	-82	0	23 112[a]	12.12
Fusiform gyrus	-42	-46	-26	23 102[a]	8.87	-36	-38	-24	23 112[a]	7.84
Fusiform gyrus	38	-48	-20	23 102[a]	8.66	34	-44	-20	23 112[a]	8.84
Intraparietal Sulcus						-20	-70	56	23 112[a]	5.23
Intraparietal Sulcus	30	-68	34	23 102[a]	4.48	22	-72	52	23 112[a]	6.20
Thalamus	28	-28	-2	23 102[a]	5.62					
Thalamus	-24	-30	2	23 102[a]	4.78					
Hippocampal gyrus	-24	-24	-10	23 102[a]	6.77					
Hippocampal gyrus	22	-24	-10	23 102[a]	6.52					
Amygdala	-26	2	-22	23 102[a]	6.70					
Amygdala	22	-6	-16	23 102[a]	5.57					
Anterior Insula	-32	28	4	23 102[a]	4.03					
Anterior Insula	28	26	-4	2 064[b]	3.89					
Orbitofrontal Cortex	-38	26	-16	23 102[a]	3.62					
Orbitofrontal Cortex	32	30	-16	2 064[b]	5.88					

Brain region	CG X	CG Y	CG Z	CG kE	CG t value	NB X	NB Y	NB Z	NB kE	NB t value
Middle Frontal Gyrus	-38	16	22	23 102[a]	4.80					
Middle Frontal Gyrus	40	10	30	2 064[b]	5.66					
Putamen	24	6	6	23 102[a]	4.20					
Supplementary Motor Area	12	6	68	420[c]	3.96					
Anterior Cingulate Cortex	10	16	44	420[c]	4.70					
Deactivations										
Anterior Cingulate Cortex						10	38	2	2 686[a]	5.63
Medial Prefrontal Cortex						24	58	10	2 686[a]	5.36
Superior Temporal Gyrus						58	-22	8	1 447	5.04
Superior Temporal Gyrus						-46	-12	4	394	4.64
Angular Gyrus						48	-56	38	574	5.56

Note. CG = complicated grief; NB = non-bereaved control group, x,y,z = peak MNI coordinates; kE = Cluster extent in voxels; [a,b,c]= part of the same cluster..

Negative valence pictures > fixation cross

Both groups showed significant activation of the visual cortex and the fusiform gyri while watching the negative valence pictures versus the fixation cross. The NB group also showed brain activation of the anterior part of the left middle frontal gyrus.

Both groups showed significant deactivation of areas of the default mode network (i.e., ventromedial prefrontal and posterior cingulate cortices, precuneus, and angular gyrus), while the NB group also showed deactivation of the right dorsolateral prefrontal cortex and temporal lobe (see Table SM3).

Table S3. Brain regions showing significant activation or deactivation associated with the processing of unpleasant/negative valence pictures.

Brain region	CG					NB				
	X	Y	Z	kE	t value	X	Y	Z	kE	t value
Negative > Fixation Cross										
Activations										
Occipital lobe	-20	-100	12	8 347[a]	6.94	-38	-82	0	18 473[a]	12.65
Occipital lobe	14	-90	6	8 347[a]	6.37	14	-90	-4	18 473[a]	13.16
Fusiform gyrus	36	-50	-18	8 347[a]	7.08	40	-62	-8	18 473[a]	10.82
Fusiform gyrus	-36	-52	-12	8 347[a]	5.47	-36	-52	-12	18 473[a]	9.43
Middle Frontal Gyrus						-52	40	14	459	5.15
Deactivations										
Anterior Cingulate Cortex	0	30	-2	512[a]	4.51	10	36	18	7 575[a]	5.29
Medial Prefrontal Cortex						8	42	2	7 575[a]	5.78
Posterior Cingulate /Precuneus	2	-26	30	696	4.32	10	-66	38	4 577[b]	8.38
Dorsolateral Prefrontal Cortex						26	38	38	7 575[a]	7.88
Angular Gyrus	64	-52	36	512	5.82	46	-58	46	2 297[a]	7.79
Angular Gyrus						-50	-60	46	1 023	6.27
Temporal Lobe						42	-4	-20	2 079	5.72

Note. CG = complicated grief group; NB = non-bereaved control group x,y,z = peak MNI coordinates; kE = Cluster extent in voxels.

Positive valence pictures > fixation cross

Both groups showed significant activation of the occipital cortex and fusiform gyri during the viewing of positive pictures versus the fixation cross, with deactivation of the posterior cingulate cortex and precuneus. Specifically, the NB group showed deactivation of the anterior cingulate

cortex, medial and dorsolateral prefrontal cortices, superior and inferior temporal cortices, angular giri, and cerebellum (see Table SM4).

Table S4. Brain regions showing significant activation, deactivation, or between-group differences associated with the processing of pleasant/positive valence pictures.

Brain region	CG					NB				
	X	Y	Z	kE	t value	X	Y	Z	kE	t value
Positive > Fixation Cross										
Activations										
Occipital lobe	-20	-100	12	7 959[a]	7.02	-10	-92	-2	16 009[a]	11.12
Occipital lobe	26	-90	8	7 959[a]	6.22	24	-90	6	16 009[a]	11.57
Fusiform gyrus	36	-52	-20	7 959[a]	6.63	40	-64	-6	16 009[a]	8.63
Fusiform gyrus	-36	-52	-14	7 959[a]	5.74	-36	-52	-14	16 009[a]	8.77
Deactivations										
Anterior Cingulate Cortex						4	38	24	3 659[a]	4.82
Medial Prefrontal Cortex						10	40	2	3 659[a]	4.73
Posterior Cingulate/Precuneus	4	-32	46	1 054	4.98	-8	-66	36	5 343	7.82
Superior Temporal Cortex						-48	-14	6	1 177	5.15
Superior Temporal Cortex						60	-30	-4	1 376	6.50
Inferior Temporal Cortex						50	-6	-34	1 018	6.12
Inferior Temporal Cortex						-50	-8	-36	389	5.15
Cerebellum						-40	-70	-38	562	5.66
Angular Gyrus						50	-56	40	1 955	8.16
Angular Gyrus						-48	-62	44	1 882	7.21
Dorsolateral Prefrontal Cortex						-28	28	36	531	5.18
Dorsolateral Prefrontal Cortex						26	42	38	3 659[a]	5.86

Note. CG = complicated grief group; NB = non-bereaved control group x,y,z = peak MNI coordinates; kE = Cluster extent in voxels.
